# Endoscopic removal of unusual gastric impaction of 30 keys and magnets in a schizophrenic patient: a rare case report

**DOI:** 10.1093/jscr/rjag175

**Published:** 2026-03-17

**Authors:** Abdisalam Ismail Hassan, Shafie Adan Dirir, Ibrahim Mohamed Abdi, Zakarie Said Dirir, Sadik Jamac Osman, Nuradin Mohamed Nur

**Affiliations:** Faculty of Medicine and Health Sciences, Jamhuriya University of Science and Technology, Mogadishu, Somalia; General Surgery Department, Shafi Hospital, Mogadishu, Somalia; General Surgery Department, Shafi Hospital, Mogadishu, Somalia; General Surgery Department, Shafi Hospital, Mogadishu, Somalia; General Surgery Department, Shafi Hospital, Mogadishu, Somalia; General Surgery Department, Mogadishu Somali-Türkiye Recep Tayyip Erdoğan Training and Research Hospital, Mogadishu, Somalia

**Keywords:** foreign body ingestion, schizophrenia, gastric impaction, endoscopic retrieval, keys, magnets, case report

## Abstract

Foreign body ingestion is a common emergency, but intentional ingestion among psychiatric patients often involves multiple and unusual objects that create significant diagnostic and therapeutic challenges. We describe a 35-year-old man with an 11-year history of untreated schizophrenia who presented with recurrent vomiting, abdominal pain, and abdominal distension. Abdominal radiography revealed a metallic mass in the stomach, and endoscopy identified numerous metallic objects, including 30 keys and 2 magnets, with minor mucosal ulcerations. All items were successfully removed using flexible endoscopy, and follow-up imaging confirmed complete clearance. The patient subsequently underwent psychiatric evaluation, and repeat endoscopy the next day showed normal findings. This case emphasizes the heightened risk of recurrent ingestion in psychiatric populations, particularly when magnets or sharp objects are involved, which may result in ulceration or perforation. Early recognition, minimally invasive retrieval, and appropriate psychiatric intervention are essential to preventing complications and reducing recurrence.

## Introduction

Intentional foreign body ingestion is a common emergency presentation encountered in emergency departments worldwide and is often challenging to manage, requiring significant time and resources [1, 2]. In adults, the most common cause of esophageal obstruction is food bolus impaction, typically meat, at the site of a preexisting stricture or ring, while in children, coins represent the most frequently ingested objects [1]. High-risk groups include children and adolescents, individuals with psychiatric or cognitive disorders, and those with alcohol or illicit drug abuse, with foreign body ingestion reported more often in males, at an estimated male-to-female ratio of 1.5:1 [1, 3].

Overall, 80%–90% of ingested foreign bodies pass spontaneously, while 10%–20% require endoscopic removal, and about 1% necessitate surgical intervention [4].

Here, we report a rare case of a schizophrenic patient who ingested 30 metallic keys along with two magnetic objects, successfully managed with flexible endoscopic retrieval. This case underscores the importance of a multidisciplinary approach involving gastroenterology, surgery, and psychiatry in the management of complex foreign body ingestion.

## Case presentation

A 35-year-old male with a known history of schizophrenia, untreated for the past 11 years and without any other chronic medical conditions, presented to our emergency department with recurrent episodes of vomiting, abdominal pain, and progressive abdominal distension.

**Figure 1:**
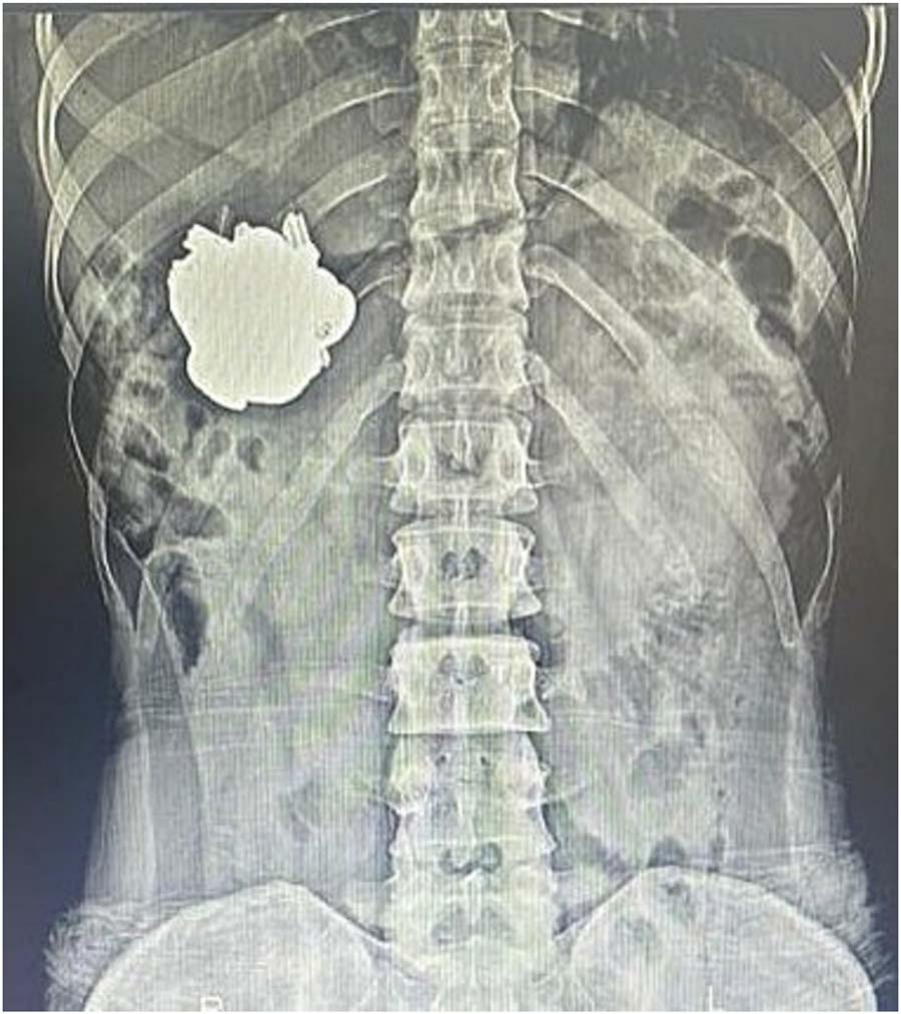
Plain abdominal X-ray (anteroposterior view) showing a large metallic foreign body projected over the left upper quadrant, corresponding to the stomach. The object has well-defined borders and dense radiopacity, consistent with an ingested metallic component.

**Figure 2:**
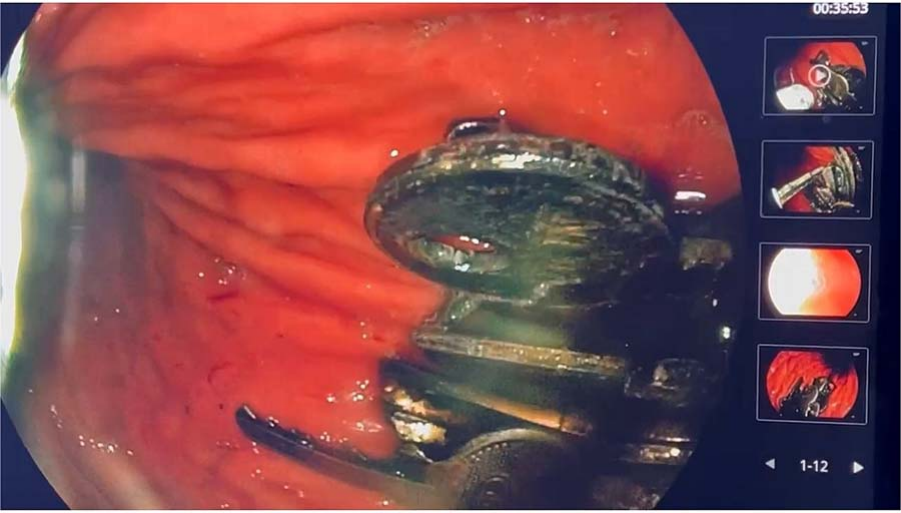
Flexible upper gastrointestinal endoscopy showing multiple metallic foreign bodies consistent with keys lodged within the gastric mucosa, associated with minor ulcerations in the gastric cardia and body.

An emergency abdominal X-ray demonstrated a metallic mass localized to the left gastric region (Fig. 1). Preoperative laboratory investigations were unremarkable. Subsequent upper gastrointestinal endoscopy revealed multiple metallic foreign bodies—identified as keys—within the gastric lumen. The gastric mucosa showed minor ulcerations involving the cardia and body (Fig. 2).

**Figure 3:**
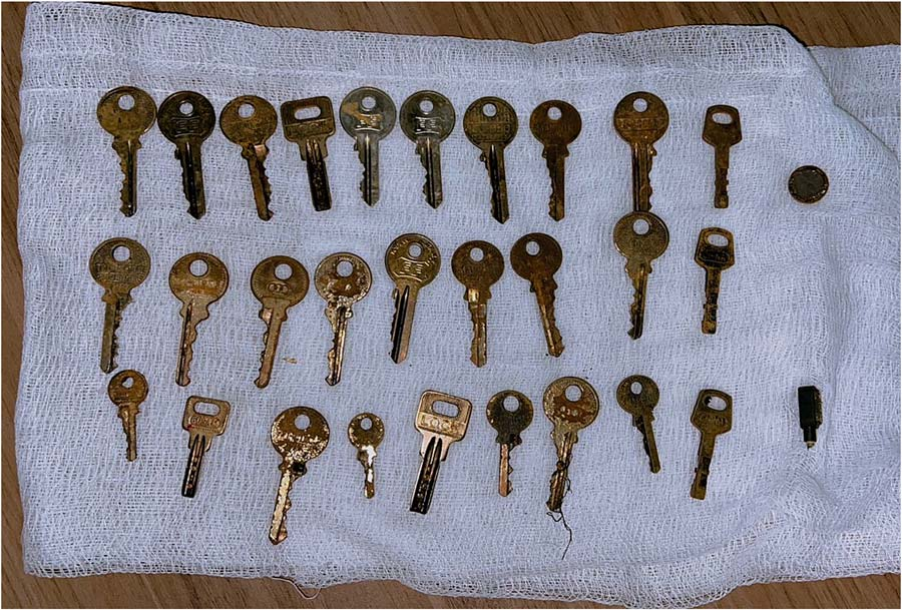
Gross photograph showing multiple metallic foreign bodies (keys and small metallic objects) laid out on gauze following endoscopic retrieval from the stomach.

Using flexible endoscopy, we successfully removed the objects, extracting a total of 30 keys and 2 magnetic metallic bodies (Fig. 3). Retrieval was performed using a flexible gastroscope with a combination of rat-tooth forceps and a retrieval basket, with an overtube used to protect the esophagus and facilitate repeated extractions. Objects were removed sequentially under direct visualization. A post-procedure abdominal X-ray confirmed complete clearance, with no residual foreign bodies. The patient was admitted for observation in the inpatient unit and subsequently referred to the psychiatric department for further evaluation. A repeat endoscopy was performed 24 h later to exclude overlooked residual foreign bodies and to reassess the previously ulcerated gastric mucosa, given the unusually large number of ingested objects and the patient’s unreliable history.

## Discussion

Foreign body ingestion is common worldwide. Adults usually present with food bolus impaction, while coins are most frequent in children [1, 2]. High-risk groups include children, psychiatric patients, and those abusing alcohol or drugs, with a male predominance of 1.5:  1 [3]. Our patient belonged to a high-risk psychiatric group and had a history of schizophrenia, a condition commonly associated with intentional or repetitive foreign body ingestion.

Common foreign bodies vary by age: coins, batteries, and toys in children versus bones and dentures in adults [1]. Impaction most often occurs in the upper esophagus, followed by the middle esophagus, stomach, duodenum, and the ileocecal junction [1]. In our case, the foreign bodies were impacted in the stomach, causing abdominal pain, distension, and vomiting rather than the classic esophageal symptoms of odynophagia or dysphagia. [5]

Most foreign bodies (80%–90%) pass spontaneously; 10%–20% require endoscopy, and <1% need surgery [4]. Complications vary by type—button batteries can cause burns, fish bones lead to perforation, and magnets may cause necrosis or fistulas [6–9]. Although the exact duration of gastric retention was unknown, early hospital presentation and prompt endoscopic intervention may have contributed to the limited mucosal injury observed.

Radiographs remain first-line imaging for suspected metallic foreign body ingestion [1], while computed tomography is reserved for radiolucent objects or when perforation is suspected [10]. Endoscopy serves both diagnostic and therapeutic roles and is the preferred modality for retrieval because of its high success rate (>95%) and lower complication risk compared with rigid endoscopy [11]. Surgical intervention is generally reserved for failed endoscopic removal or in the presence of complications such as perforation or obstruction [12]. In our case, abdominal radiography localized the metallic mass to the stomach, and flexible endoscopy enabled complete retrieval of 30 keys and 2 magnets without the need for surgery. This highlights the effectiveness of minimally invasive management, even in cases involving multiple, high-risk metallic foreign bodies.

## Conclusion

This case illustrates an exceptionally rare presentation of massive gastric foreign body ingestion in a patient with schizophrenia, involving 30 keys and 2 magnets. Early diagnosis with radiography, followed by successful flexible endoscopic retrieval, allowed for complete clearance without surgical intervention. The outcome underscores the importance of prompt recognition, minimally invasive management, and appropriate psychiatric assessment and follow-up as important components of care in high-risk patients.

## Data Availability

The data supporting the findings of this study are available from both corresponding authors upon reasonable request.
